# Versorgung älterer Patienten durch Gemeindenotfallsanitäter: Vergleich der Einsätze im Pflegeheim und in der Häuslichkeit

**DOI:** 10.1007/s00063-021-00863-8

**Published:** 2021-09-16

**Authors:** Insa Seeger, Ulf Günther, Guido Schmiemann, Falk Hoffmann

**Affiliations:** 1 Oldenburger Forschungsnetzwerk Notfall- und Intensivmedizin, Carl von Ossietzky Universität Oldenburg, Oldenburg, Deutschland; 2 Universitätsklinik für Anästhesiologie | Intensivmedizin | Notfallmedizin | Schmerztherapie, Klinikum Oldenburg AöR, Oldenburg, Deutschland; 3 Abteilung Versorgungsforschung, Institut für Public Health und Pflegeforschung (IPP), Universität Bremen, Bremen, Deutschland; 4 Health Sciences Bremen, Universität Bremen, Bremen, Deutschland; 5 Department für Versorgungsforschung, Carl von Ossietzky Universität Oldenburg, Ammerländer Heerstr. 140, 26129 Oldenburg, Deutschland

**Keywords:** Pflegebedürftige, Versorgungsforschung, Notfallversorgung, Rettungsdienst, Ambulante Versorgung, People in need of care, Health services research, Emergency care, Emergency medical services, Ambulatory care

## Abstract

**Hintergrund:**

Notaufnahmen und Rettungsdienste werden zunehmend durch nicht vital bedrohlich erkrankte Patienten belastet. Ein großer Anteil der Rettungsdiensteinsätze entfällt auf ältere Menschen. Um eine unnötige Disponierung höherwertiger Rettungsmittel zu reduzieren, wurde das Modellprojekt Gemeindenotfallsanitäter (G-NFS) entwickelt.

**Ziel der Arbeit:**

Ziel dieser Arbeit war es herauszufinden, ob sich spezifische Einsatzschwerpunkte des G‑NFS in der Versorgung älterer Menschen in häuslicher Umgebung und im Pflegeheim zeigen.

**Material und Methoden:**

Es handelt sich um eine retrospektive Beobachtungsstudie auf Basis der Einsatzdokumentation vom 01.07.2019 bis zum 30.06.2020. Es wurden G‑NFS Einsätze bei älteren Menschen (≥ 65 Jahre) analysiert, unterteilt nach den Einsatzorten Pflegeheim oder Häuslichkeit.

**Ergebnisse:**

Es wurden 2358 Protokolle ausgewertet (Durchschnittsalter: 80,8 Jahre; 52,9 % weiblich). Vor Ort wurden 55 % der Patienten versorgt. Die durchgeführten Maßnahmen umfassten Beratungsgespräche (79,4 %), Hilfe bei Selbstmedikation (16,7 %) und Medikamentengabe (23,2 %). Auf Pflegeheimbewohner entfielen 329 (14,0 %) Einsätze. Maßnahmen in Bezug auf Urindauerkatheter wurden häufiger bei Pflegeheimbewohnern als in der Häuslichkeit durchgeführt (32,2 % bzw. 5,7 % aller Einsätze). Bei Patienten mit Katheterproblemen erfolgte im Vergleich zu anderen Einsätzen nahezu immer eine Versorgung vor Ort (84,3 % bzw. 52,2 %).

**Diskussion:**

Durch G‑NFS können die Mehrzahl der älteren Patienten ambulant versorgt und andere Ressourcen entlastet werden. Allerdings führen G‑NFS auch Maßnahmen durch, die im Verantwortungsbereich der hausärztlichen Versorgung liegen, und gleichen somit strukturelle Defizite in der medizinisch-pflegerischen Versorgung aus.

**Zusatzmaterial online:**

Die Onlineversion dieses Beitrags (10.1007/s00063-021-00863-8) enthält die Tabellen S1–S3.

## Abstract (en)

**Background:**

Emergency departments and emergency services are increasingly burdened by non-emergency patients. A substantial proportion of these is represented by older people. The project “community emergency paramedic” (“Gemeindenotfallsanitäter” [G-NFS]) was initiated to prevent unnecessary use of emergency services.

**Objective:**

To identify specific utilizations of the G‑NFS services by older people at home and in nursing homes.

**Material and methods:**

Retrospective analysis of the assignment protocols from July 2019 through June 2020. Only data from patients aged ≥ 65 years were included. Data were grouped into whether patients lived on their own or in nursing homes.

**Results:**

A total of 2358 protocols of older patients (mean age 80.8 years; 52.9% female) were evaluated and 55% of patients were treated on-site. The most frequently used measures by G‑NFS were counselling (79.4%), aid in self-medication (16.7%) and administration of medication (23.2%). Of the G‑NFS assignments 329 (14.0%) were carried out for nursing home residents. Measures related to urine catheter complications were more frequently performed in nursing home residents than in patients who lived at home (32.2% vs. 5.7%). Compared to other emergency cases, patients with catheter-related complications were most often treated at the scene (84.3% vs. 52.2%).

**Conclusion:**

The G‑NFS enabled the majority of patients to be treated on-site, thus saving resources of emergency services and hospitals; however, the G‑NFS also performed measures that were normally the responsibility of general practitioners. This possibly highlights structural deficits in the medical and nursing care of older people.

## Hintergrund und Fragestellung

Im Zuge des demographischen Wandels ändern sich auch Bedarf und Inanspruchnahme von Versorgungsleistungen. So nimmt mit steigender Lebenserwartung auch der Bedarf an akuter medizinischer Versorgung zu. Dies zeigt sich sowohl in der Inanspruchnahme von Notaufnahmen [1, 2] wie auch bei Rettungsdiensteinsätzen [3]. Dadurch hat sich auch das Einsatzspektrum im Rettungsdienst gewandelt: Während traumatologische Einsatzgründe abnehmen, ist bei älteren Patienten eine deutliche Steigerung internistischer Erkrankungen zu beobachten [4, 5].

Notaufnahmebesuche von Pflegeheimbewohnern spielen dabei eine nicht zu unterschätzende Rolle [6], wobei ein Teil der Transporte als vermeidbar eingeschätzt wird [7, 8]. Häufig werden Pflegeheimbewohner allein aus haftungsrechtlichen Gründen in Notaufnahmen transportiert [9]. Dies trifft aber auch für ältere Patienten in der Häuslichkeit zu. Nicht alle in den Notaufnahmen vorstelligen älteren Patienten werden jedoch stationär aufgenommen [10].

Um dem steigenden Einsatzaufkommen durch nicht vital bedrohliche Notfälle zu begegnen, haben 4 Rettungsdienstträgerschaften im Oldenburger Land das Modellprojekt des Gemeindenotfallsanitäters (G-NFS) entwickelt [11, 12]. Ziel dieser Studie war es, die Einsätze des G‑NFS für ältere Menschen im Pflegeheim und der Häuslichkeit zu analysieren.

## Studiendesign und Untersuchungsmethoden

### Setting und Implementierung des Gemeindenotfallsanitäters

Im Rahmen eines Pilotprojekts werden seit dem 01.01.2019 rund um die Uhr G‑NFS in 4 niedersächsischen Landkreisen mit insgesamt rund 600.000 Einwohnern als zusätzliches Rettungsmittel eingesetzt, wenn es sich nicht um bereits in der Leitstelle identifizierte Notfälle handelt bzw. keine ärztliche Intervention oder ein Transport erwartet wird [11].

G‑NFS fahren alleine und ohne Sonderrechte zum Einsatzort. Wenn sich ein G‑NFS in der Nähe des Einsatzorts befindet, kann er von der Leitstelle zusätzlich als sog. First Responder alarmiert werden, um Wartezeiten auf den Rettungswagen (RTW) zu überbrücken. Bei den G‑NFS handelt es sich derzeit um 25 erfahrene Notfallsanitäter, die eine zusätzliche 3‑monatige Weiterbildung mit theoretischen und praktischen Unterrichtsphasen (u. a. Praktika in Hausarztpraxis, chirurgischer Ambulanz und urologischer Einrichtung) absolviert haben. Neben fachlichen, methodischen, sozialen und personalen Kompetenzen sind auch die von den ärztlichen Leitern der beteiligten Rettungsdienste freigegebenen Algorithmen integrativer Bestandteil der Weiterbildung [11]. Nach Erstkontakt, Anamnese und Untersuchung können somit erste pflegerisch-medizinische Maßnahmen vor Ort durchgeführt und bei Bedarf über die Notwendigkeit der weiteren Versorgung entschieden werden. Ferner besteht die Möglichkeit, jederzeit eine ärztliche Mitbeurteilung durch die Telemedizinzentrale der Universitätsklinik für Anästhesiologie (AINS) im Klinikum Oldenburg zu erhalten. Sollte vor Ort doch ein Notfall identifiziert werden, werden die lokalen Algorithmen für Notfallsanitäter angewendet und weitere Rettungsmittel nachgefordert.

### Datenbasis und Studienpopulation

Die Studie wurde als retrospektive Beobachtungsstudie durchgeführt. Im Zeitraum vom 01.07.2019 bis zum 30.06.2020 wurden die Daten aller Patienten eingeschlossen, die 65 Jahre und älter waren und von den G‑NFS versorgt wurden. Datenbasis bildeten die G‑NFS-Einsatzprotokolle, die zusätzlich zum DIVI-Einsatzprotokoll ausgefüllt wurden.

Die G‑NFS-Einsatzprotokolle werden monatlich von den Rettungsdiensten auf einem gesicherten Weg an die Universität Oldenburg übermittelt. Die Protokolle wurden mithilfe der Erfassungssoftware Teleform (Electric Paper Informationssysteme, Lüneburg, Deutschland) Version 16.5 eingelesen. Von der Erfassungssoftware nicht korrekt erfasste Auswahl- und Freitextfelder wurden manuell korrigiert.

Ein positives Ethikvotum der Medizinischen Ethikkommission der Universität Oldenburg liegt vor (Votums-Nr. 2019-030).

### Einsatzprotokoll und erfasste Variablen

Im G‑NFS-Einsatzprotokoll wurden u. a. Geburtsjahr, Geschlecht und ob es sich um einen Pflegeheimpatienten handelt, erfasst (Tab. S1). Die Behandlungspriorität wurde in Anlehnung an den von der Software IVENA (webbasierter interdisziplinärer Versorgungsnachweis, um sich in Echtzeit über die aktuellen Versorgungsmöglichkeiten in den Krankenhäusern zu informieren; [13]) verwendeten Patientenzuweisungscode (PZC) eingestuft. Die G‑NFS dokumentierten die durchgeführten Maßnahmen, die Inanspruchnahme von Telemedizin, die Notwendigkeit eines Transports, welche Empfehlungen die Patienten zur weiteren Versorgung von den G‑NFS erhielten und ob eine Kontaktaufnahme des G‑NFS zum Hausarzt oder kassenärztlichem Bereitschaftsdienst nach dem Einsatz erfolgte. Die Angaben im Protokoll waren als Einfach- oder Mehrfachantwort zu beantworten, in den Kategorien „Maßnahmen“ und „Empfehlungen“ bestand zusätzlich die Möglichkeit, ein Freitextfeld zu nutzen. In der Kategorie „Maßnahmen“ wurden die Antwortmöglichkeiten „Entfernung Dauerkatheter“ und „Wiederanlage Dauerkatheter“ zu „Katheterversorgung“ sowie entsprechende Freitextangaben zusammengefasst. Hierbei handelt es sich ausschließlich um die Versorgung von transurethralen Urindauerkathetern. Auch die unterschiedlichen Maßnahmen in Bezug auf die Medikationsgabe sowie „Sonstige Maßnahmen“ („Hilfe beim Inhalieren“, „Kompressionsstrümpfe“, „Urinstix“ und „Sonstiges“) wurden zusammengefasst.

### Statistische Analyse

Die Daten wurden deskriptiv analysiert. Die Auswertungen wurden unterteilt nach dem Einsatzort Pflegeheim sowie der Häuslichkeit, wobei Unterschiede mittels χ^2^-Test analysiert wurden. Für die Analysen wurden zusätzlich Kategorien für die stetigen Variablen „Alter“ (65–74 Jahre, 75–84 Jahre und 85 Jahre und älter) und „Einsatzdauer“ gebildet (weniger als 30 min, 30–59 min, 60–89 min, 90 min und mehr).

Die Auswertung erfolgte mithilfe der Statistiksoftware IBM SPSS Statistics for Windows (Version 26.0, SPSS Inc., Chicago, IL, USA) und SAS für Windows Version 9.4 (SAS Institute Inc, Cary, NC, USA).

## Ergebnisse

### Baseline-Charakteristika

Von den insgesamt 4390 durch G‑NFS dokumentierten Einsätzen entfielen 2358 (53,7 %) auf Personen im Mindestalter von 65 Jahren. Diese waren durchschnittlich 80,8 Jahre alt und etwa die Hälfte war weiblich (52,9 %). Die Mehrzahl der Einsätze wurde als nicht dringlich eingestuft und die durchschnittliche Einsatzzeit lag bei etwa einer Stunde (Tab. 1).

**Table 1:** 

	Gesamt (*n* = 2358; 100 %)	Häuslichkeit (*n* = 2029; 86,0 %)	Pflegeheim (*n* = 329; 14,0 %)	*p*-Wert
*Alter in Jahren, Mittelwert (SD)*	80,8 (7,8)	80,3 (7,7)	83,6 (7,4)	–
*Altersgruppen (n* *=* *2358)*				< 0,001
65–74 Jahre	23,3 %	25,2 %	11,9 %	
75–84 Jahre	43,5 %	43,5 %	43,8 %	
85+ Jahre	33,2 %	31,4 %	44,4 %	
*Geschlecht (n* *=* *2320)*				< 0,001
Männer	47,1 %	44,8 %	61,0 %	
Frauen	52,9 %	55,2 %	39,0 %	
*First Responder (n* *=* *2358)*	7,4 %	7,5 %	6,4 %	0,456
*Dringlichkeit des Einsatzes (n* *=* *2294)*				0,145
PZC 0	56,0 %	55,7 %	57,8 %	
PZC 1	8,6 %	9,1 %	5,9 %	
PZC 2	20,4 %	20,7 %	18,8 %	
PZC 3	15,0 %	14,6 %	17,5 %	
*Einsatzdauer in min, Mittelwert (SD)*	64,1 (29,8)	64,5 (30,9)	61,6 (21,9)	–
*Einsatzdauer in Gruppen (n* *=* *2319)*				< 0,001
< 30 min	5,6 %	5,7 %	4,9 %	
30–59 min	37,9 %	38,1 %	36,7 %	
60–89 min	40,8 %	39,5 %	49,1 %	
90+ min	15,8 %	16,8 %	9,3 %	

Insgesamt 329 Einsätze entfielen auf Pflegeheimbewohner (14,0 %). Diese waren im Vergleich zu in der Häuslichkeit Versorgten etwas älter (83,6 bzw. 80,3 Jahre) und zu einem deutlich höheren Anteil männlich (61,0 % bzw. 44,8 %). Dringlichkeit und Einsatzzeiten unterschieden sich nicht zwischen diesen beiden Gruppen.

### Maßnahmen des Gemeindenotfallsanitäters

Insgesamt wurden am häufigsten Beratungen dokumentiert (79,4 %). Hilfe bei der Selbstmedikation als auch Gaben ausgewählter Medikamente waren ebenfalls häufig (Tab. S2). Seltener wurden Maßnahmen in Bezug auf die Versorgung von Urindauerkathetern (9,4 %) sowie Wundversorgung durchgeführt (3,5 %). Der deutlichste Unterschied nach Alter und Geschlecht zeigte sich bei Problemen mit Dauerkathetern, die insgesamt häufiger bei Männern dokumentiert wurden (17,2 % bzw. 2,3 %).

Der größte Unterschied zwischen Einsätzen im Pflegeheim und in der Häuslichkeit betraf ebenfalls Dauerkatheter. Insgesamt wurden solche Maßnahmen bei einem Drittel der versorgten Pflegeheimbewohner und damit etwa 6‑mal häufiger als in der Häuslichkeit durchgeführt (32,2 % bzw. 5,7 %). Bei Männern im Pflegeheim wurden bei nahezu jedem zweiten Einsatz Maßnahmen in Bezug auf Dauerkatheter ergriffen (49,5 % bzw. 5,5 % bei Frauen). Wundversorgungen spielen im Pflegeheim ebenfalls eine etwas größere Rolle als bei Einsätzen in der Häuslichkeit (6,4 % bzw. 3,1 %). Entsprechend wurden Beratungen und Maßnahmen in Bezug auf die Medikation bei Einsätzen im Pflegeheim seltener durchgeführt.

### Weiterbehandlung und Empfehlungen des Gemeindenotfallsanitäters

Insgesamt war bei mehr als der Hälfte der Einsätze eine Versorgung vor Ort möglich (Tab. 2). Hausärzte oder der KV-Notdienst wurden in 16 % der Fälle hinzugezogen. Eine Vorstellung in der Notaufnahme bzw. beim Hausarzt wurde bei jeweils etwa einem Drittel der Einsätze empfohlen. Die Möglichkeit, einen Arzt mittels Telemedizin zuzuschalten, wurde nur selten genutzt.

**Table 2:** 

	Gesamt (*n* = 2358), in %	Häuslichkeit (*n* = 2029), in %	Pflegeheim (*n* = 329), in %	*p*-Wert
*Nutzung von Telemedizin (n* *=* *2358)*	1,2	1,1	1,5	0,491
*Konsultation Hausarzt/KV-Notdienst (n* *=* *2358)*	16,0	16,7	11,6	0,018
*Empfehlung weiterer Versorgung (n* *=* *2358)*				
Vorstellung in Notaufnahme	37,9	38,5	34,0	0,119
Vorstellung beim Hausarzt	36,2	38,0	25,2	< 0,001
Vorstellung KV-Notdienst	4,3	4,8	0,9	0,001
*Transport (n* *=* *2318)*				< 0,001
KTW	19,6	19,2	22,5	
RTW	18,7	19,1	16,4	
Sonstiger	6,5	7,3	1,2	
Keiner	55,2	54,5	59,9	

Bei der Weiterbehandlung in Abhängigkeit des Einsatzorts gab es nur wenige Unterschiede (ausschließliche Versorgung vor Ort 59,9 % bzw. 54,5 %). Hausärzte wurden etwas seltener im Pflegeheim kontaktiert (11,6 % bzw. 16,7 %) und bei Pflegeheimeinsätzen wurde ebenfalls seltener eine Vorstellung beim Hausarzt empfohlen (25,2 % bzw. 38,0 %).

### Probleme mit Dauerkathetern im Fokus

Wie bereits gezeigt wurden bei 9,4 % der Einsätze Maßnahmen in Bezug auf Dauerkatheter durchgeführt (*n* = 221; bei 84,2 % erfolgte die Wiederanlage eines Dauerkatheters). Diese unterschieden sich in zahlreichen Charakteristika deutlich von anderen Einsätzen (Tab. S3). Knapp die Hälfte dieser Einsätze erfolgte im Pflegeheim (48,0 % bzw. 10,4 % bei anderen Einsätzen) und sie betrafen deutlich häufiger Männer (87,0 % bzw. 43,0 %). Sie waren zudem als weniger dringlich eingeschätzt und bei ihnen wurde seltener der Hausarzt kontaktiert bzw. eine Weiterbehandlung empfohlen. Bei insgesamt 84,3 % der Einsätze, bei denen Maßnahmen in Bezug auf Dauerkatheter (Spülung, Entfernung, Wiederanlage) durchgeführt wurden, erfolgte eine Versorgung vor Ort (52,2 % bei anderen Einsätzen). Die Einsatzdauern unterschieden sich nicht.

**Figure 1:**
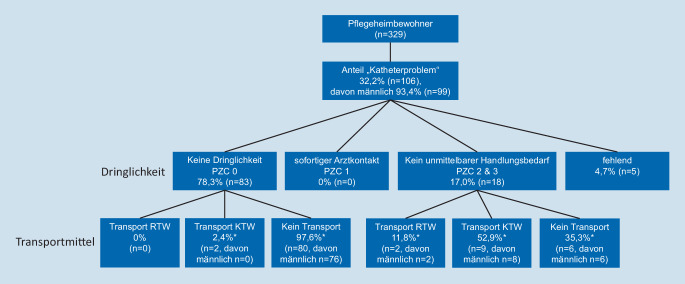

Die nähere Betrachtung der Katheterversorgung von Pflegeheimbewohnern wird in Abb. 1 dargestellt. 93,4 % der Pflegeheimbewohner mit Katheterproblemen waren männlich. Als nicht dringlich zu versorgen (PZC 0) wurden 78,3 % eingestuft, davon wurden 97,6 % vor Ort versorgt. Kein unmittelbarer Handlungsbedarf, aber weitere Diagnostik war bei 17,0 % erforderlich. Bei knapp zwei Drittel (64,7 %) der Patienten, bei denen kein unmittelbarer Handlungsbedarf (PZC 2 und 3) bestand, wurde ein Rettungsmittel nachgefordert, um einen Transport ins Krankenhaus durchzuführen.

## Diskussion

Insgesamt wurden etwa 2400 Einsätze der G‑NFS bei Personen im Mindestalter von 65 Jahren ausgewertet, von denen 14,0 % im Pflegeheim stattfanden. Bei mehr als der Hälfte der Fälle konnten die Patienten vor Ort versorgt werden, auch die Einsatzzeit von etwa einer Stunde unterschied sich nicht in Abhängigkeit davon, ob Patienten im Pflegeheim oder in der Häuslichkeit versorgt wurden. Auffällig ist jedoch, dass bei einem Drittel der Einsätze im Pflegeheim Maßnahmen in Bezug auf Dauerkatheter durchgeführt wurden, bei männlichen Bewohnern sogar bei jedem zweiten Einsatz. Solche Maßnahmen spielen bei Einsätzen in der Häuslichkeit hingegen keine nennenswerte Rolle.

### Anzahl von Transporten

Während bei rund 38 % der versorgten Patienten vom G‑NFS ein Rettungsmittel nachgefordert wurde, konnte bei 55 % der Patienten auf einen Transport verzichtet werden. Damit verbundene Ressourcen in Rettungsdienst und Notaufnahmen konnten folglich eingespart werden und die älteren Patienten verblieben in ihrer vertrauten Umgebung. Dies ist von erheblicher Bedeutung, da verschiedene Studien zeigen, dass ältere Notfallpatienten nicht nur einen deutlich höheren Arbeitsaufwand für die Notaufnahmen bedeuten und häufiger stationär aufgenommen werden, sondern auch nach Entlassung eine höhere Rate an negativen gesundheitlichen Folgen, wie z. B. Verschlechterung der körperlichen oder kognitiven Fähigkeiten, haben [1, 10, 14]. Besonders bei Pflegeheimbewohnern führt der Transport in ein Krankenhaus nicht selten zu einer längeren stationären Versorgung und zu einer Verschlechterung des Gesundheitszustands [15]. Darüber hinaus zeigte eine Befragung von Rettungsdienstpersonal, dass Krankenhaustransporte z. B. bei Katheterproblemen, Exsikkose oder Stürzen vermeidbar wären, aber aufgrund gesetzlicher Rahmenbedingungen einen Transport durchgeführt werden muss [16]. Die dadurch bedingte Frustration der RTW-Besatzung könnte durch den Einsatz von G‑NFS ggf. gemindert werden.

### Gemeindenotfallsanitäter als Alternative und Brücke zum Hausarzt

Einem Drittel der Patienten wurde die Vorstellung beim Hausarzt empfohlen. Nach erfolgter Anamnese und Untersuchung wurde bei 83 % der in der Häuslichkeit versorgten Patienten ein Beratungsgespräch durchgeführt, während im Pflegeheim rund 58 % beraten wurden. Der zunehmende Mangel an niedergelassenen Ärzten und die abnehmende Bereitschaft, Hausbesuche durchzuführen, wird als ein Grund für die Steigerung der Rettungsdiensteinsätze diskutiert [17, 18]. Teilweise scheinen diese Einsätze auch eher durch pflegerische als durch notfallmedizinische Aspekte begründet zu sein [19]. Somit übernimmt der Rettungsdienst gerade bei älteren Menschen Aufgaben, die der hausärztlichen Versorgung zuzuschreiben sind. Untersuchungen aus Deutschland zeigen für das Setting Pflegeheim, dass dort regelmäßige und teils präventive hausärztliche Besuche stattfinden [8, 17]. Dies ist vermutlich für ältere Patienten, die in der Häuslichkeit leben, nicht flächendeckend gegeben. Aufgrund eingeschränkter Mobilität und zunehmender Pflegebedürftigkeit sind auch diese Patienten oft nicht in der Lage, die Hausarztpraxis aufzusuchen und somit auf einen Hausbesuch angewiesen. Die eingeschränkte Verfügbarkeit hausärztlicher Versorgung könnte ursächlich für die Inanspruchnahme der Notrufnummer 112 sein.

### Katheterversorgung im Pflegeheim

Bei 9,4 % aller G‑NFS-Einsätze wurden Patienten mit Dauerkatheter versorgt, im Pflegeheim sogar bei etwa jedem dritten Einsatz. Bei 84 % der Patienten war durch die Versorgung vor Ort kein Transport in weiterführende Versorgungseinrichtungen notwendig. Insgesamt haben nach einer Studie aus Bremen und Niedersachsen 13,4 % der Pflegeheimbewohner einen Dauerkatheter, bei Männern sind es 25,3 % und bei Frauen 9,7 % [20]. Dass Männer trotzdem deutlich häufiger vom G‑NFS oder ambulant im Krankenhaus versorgt werden als Frauen, erklärt sich vermutlich durch die Politik des Pflegeheims. In vielen Einrichtungen ist das Personal angehalten, transurethrale Katheter nur bei Frauen, nicht aber bei Männern zu wechseln. Belastbare Daten zur Häufigkeit sind uns jedoch nicht bekannt. Rettungsdienstkräfte halten einen Transport ins Krankenhaus bei Katheterproblemen für potenziell vermeidbar, da sie ebenso gut ambulant zu behandeln sind [21]. Zwar ist die Katheterversorgung Bestandteil der Ausbildung von Pflegekräften und kann vom behandelnden Arzt an qualifizierte Mitarbeiter delegiert werden [22], aber fehlende Absprachen mit dem Hausarzt, heiminterne Anweisungen, mangelnde Erfahrungen und der Einsatz von unzureichend qualifiziertem Pflegepersonal führen sicherlich dazu, das Problem durch eine Alarmierung des Rettungsdienstes zu lösen. Insgesamt können Wechsel eines transurethralen Katheters durch das Pflegepersonal erfolgen, während geplante Wechsel eines suprapubischen Blasenkatheters durch Hausärzte bzw. niedergelassene Urologen durchgeführt werden könnten.

### Stärken und Schwächen

Wesentliche Stärke dieser Arbeit ist, dass insgesamt etwa 2400 und damit eine große Zahl an Einsatzdokumenten des G‑NFS aus verschiedenen Landkreisen ausgewertet werden konnten. Es ist jedoch möglich, dass nicht für alle Einsätze Protokolle ausgefüllt wurden, wobei es sich nach Abgleich verschiedener Daten im Zuge der Qualitätssicherung eher um eine geringere Anzahl handeln dürfte. Insgesamt wurden zwar mehr als 300 Einsätze im Pflegeheim dokumentiert, jedoch ist diese Zahl im Vergleich zu den Gesamteinsätzen niedrig. Eine weitere mögliche Limitation könnte die Untererfassung von Einsätzen im Pflegeheim sein, da erst ab Juli 2019 ein zusätzliches Feld zum Setting Pflegeheim ins Protokoll eingefügt wurde. Gleichzeitig sind die Maßnahmen nur sehr grob zuzuordnen, wobei jedoch in Bezug auf Dauerkatheter sowohl die Entfernung als auch die Anlage als separate Felder verfügbar sind und wir zusätzlich die Freitextangaben mitberücksichtigt haben. Allerdings liegen keine weiteren Informationen dazu vor, wie häufig es sich um planbare Katheterwechsel oder um akute Probleme bzw. um suprapubische Blasenkatheter handelte. Ebenso sind uns die Uhrzeit der Einsätze und weitere klinische Angaben (z. B. Pflegegrade, Mobilität, Demenz) nicht bekannt. Nach dem Einsatz können weitere Maßnahmen oder Transporte erfolgt sein, zu denen uns keine Informationen vorliegen. Ebenso wissen wir nicht, ob bestimmte Personen mehrfach besucht wurden, da in unseren Daten keinerlei Personenbezug vorliegt. Zu beachten ist auch, dass Einsätze des G‑NFS in einer einzelnen und vergleichsweise ländlichen Region untersucht wurden. Allerdings ist der G‑NFS ein Modellprojekt, das ausschließlich in dieser Region eingeführt wurde, und entsprechend handelt es sich um eine Vollerhebung aller Einsätze.

## Fazit für die Praxis

Insgesamt finden mehr als die Hälfte der Einsätze des G‑NFS bei Personen im Mindestalter von 65 Jahren statt, dabei jeder 7. Einsatz im Pflegeheim. Der G‑NFS ist eine Option des Leitstellendisponenten zur Versorgung nichtdringlicher Notfälle und entsprechend konnte bei 55 % ein Transport ins Krankenhaus vermieden werden. Im Setting Pflegeheim fielen besonders Probleme in der Katheterversorgung auf, wobei sich grundsätzlich die Frage stellt, ob hier die Alarmierung über die Notrufnummer 112 das Mittel der Wahl ist. Insgesamt sollten G‑NFS nicht zur Kompensation der strukturellen Defizite in der medizinischen und pflegerischen Versorgung dienen. Grundsätzlich muss diskutiert werden, inwiefern in Zeiten des zunehmenden demographischen Wandels die gesundheitliche Versorgung älterer Menschen insgesamt sichergestellt und besser koordiniert werden kann.

## Supplementary Information




