# Supplementary material for: Care of older patients by community emergency paramedics: Comparison of community-dwellers and nursing home residents
Source: Med Klin Intensivmed Notfmed. 2021 Sep 16;117(7):542–8. [Article in German] doi: 10.1007/s00063-021-00863-8 (PMC9553795; doi:10.1007/s00063-021-00863-8)
Supplement: Supplementary file 1 [file 63_2021_863_MOESM1_ESM.pdf]

Tabelle S1: Angaben GNFS-Protokoll

**Demografische Daten**	
Geburtsjahr, Geschlecht	
**Sonstiges**	
First Responder Einsatz, Patient aus Pflegeheim	Einfachauswahl
**PZC (Patientenzuweisungscode)**	
PZC 0: kein Transport<br>PZC 1: sofortige intensivmedizinische Intervention<br>PZC 2: stationäre Aufnahme wahrscheinlich<br>PZC 3: ambulante Behandlung	Einfachauswahl
**Maßnahmen**	
Beratungsgespräch, Hilfe bei Selbstmedikation oder Inhalation, Verbandwechsel/Wundversorgung, Kompressionsstrümpfe, Urinstix, Dauerkatheter anlegen/entfernen, i.v.-Zugang, Medikamentengabe	Mehrfachauswahl
**Inanspruchnahme Telemedizin**	
Wurde genutzt: nach Algorithmus, eigene Entscheidung, sonstiges<br>Wurde nicht genutzt: nicht erforderlich, kein Funkempfang, stand nicht zur Verfügung	Mehrfachauswahl
**Notwendigkeit eines Transports**	
Rettungswagen, Krankentransportwagen, Taxi/Mietwagen, Rollstuhltransport, privater Transport oder auf Verzicht auf Transport	Einfachauswahl
**Empfehlungen**	
Vorstellung: Hausarzt, KV-Bereitschaftsdienst, kinderärztlicher Notdienst, Notaufnahme, Information von Angehörigen, des ambulanten Pflegedienstes oder Krisenintervention	Mehrfachauswahl
**Kontaktaufnahme zum Hausarzt / KV-Bereitschaftsdienst durch Patienten**	
Hausarzt/KV-Bereitschaftsdienst wurde kontaktiert, Kontakt zum Hausarzt/ KV-Bereitschaftsdienst nicht ausreichend, Hausarzt/KV-Bereitschaftsdienst wurde nicht erreicht.	Mehrfachauswahl
**Kontaktaufnahme zum Hausarzt KV-Bereitschaftsdienst durch G-NFS**	
Hausarzt/KV-Bereitschaftsdienst wurde konsultiert, vergeblicher Versuch, Hausarzt/KV-Bereitschaftsdienst zu konsultieren, kein Versuch einer Konsultation	Mehrfachauswahl
**Einschätzung des GNFS zur Kategorisierung durch die Leitstelle**	
richtig, zu hoch oder zu niedrig kategorisiert, telefonische Fachberatung wäre ausreichend gewesen	Einfachauswahl

Tabelle S2: Maßnahmen bei Einsätzen von Gemeindenotfallsanitätern, gesamt und stratifiziert nach Einsatzort

	Beratung	Hilfe bei Selbst-medikation	Medikations-gabe	Dauerkatheter	Wundver-sorgung	Sonstige
Häuslichkeit (n=1.873)						
Altersgruppen						
65-74 Jahre	85,1%	19,4%	26,0%	2,5%	2,7%	40,7%
75-84 Jahre	82,7%	17,9%	23,9%	5,3%	3,3%	43,3%
85+ Jahre	81,3%	18,4%	22,3%	8,7%	3,0%	44,2%
Geschlecht						
Männer	80,6%	16,2%	22,8%	10,0%	3,3%	46,0%
Frauen	85,2%	20,6%	24,9%	1,9%	2,7%	40,3%
Gesamt	82,9%	18,4%	24,0%	5,7%	3,1%	42,9%
Pflegeheim (n=329)						
Altersgruppen						
65-74 Jahre	51,3%	0%	18,0%	30,8%	5,1%	48,7%
75-84 Jahre	59,7%	5,6%	14,6%	37,5%	5,6%	36,1%
85+ Jahre	58,9%	7,5%	22,6%	27,4%	7,5%	39,7%
Geschlecht						
Männer	53,0%	4,0%	15,0%	49,5%	5,5%	35,0%
Frauen	66,4%	8,6%	24,2%	5,5%	7,8%	45,3%
Gesamt	58,4%	5,8%	18,5%	32,2%	6,4%	39,2%
Gesamt (n=2.358)						
Altersgruppen						
65-74 Jahre	82,7%	18,0%	24,5%	4,5%	2,9%	41,3%
75-84 Jahre	79,4%	16,2%	22,6%	9,8%	3,6%	42,3%
85+ Jahre	77,1%	16,4%	22,4%	12,1%	3,8%	43,4%
Geschlecht						
Männer	75,6%	14,0%	21,4%	17,2%	3,7%	44,0%
Frauen	83,2%	19,3%	24,9%	2,3%	3,3%	40,8%
Gesamt	79,4%	16,7%	23,2%	9,4%	3,5%	42,4%

Tabelle S3: Charakteristika von Einsätzen in Abhängigkeit ob Anlage bzw. Entfernung eines Dauerkatheters durchgeführt wurde

	Dauerkatheter (n=221)	Kein Dauerkatheter (n=2.137)
Einsatzort		
Häuslichkeit	52,0%	89,6%
Pflegeheim	48,0%	10,4%
Alter in Jahren, Mittelwert (SD)	83,0 (6,7)	80,6 (7,8)
Geschlecht		
Männer	87,0%	43,0%
Frauen	13,0%	57,0%
Dringlichkeit des Einsatzes		
PZC 0	81,5%	53,4%
PZC 1	0%	9,5%
PZC 2	1,0%	22,4%
PZC 3	17,5%	14,7%
Einsatzdauer in Minuten, Mittelwert (SD)	62,9 (25,2)	64,2 (30,2)
Konsultation Hausarzt/ KV-Notdienst	6,8%	16,9%
Empfehlung weiterer Versorgung		
Vorstellung in Notaufnahme	5,9%	41,2%
Vorstellung beim Hausarzt	19,5%	37,9%
Vorstellung KV-Notdienst	0,5%	4,7%
Kein Transport erfolgt	84,3%	52,2%
